# Supplementary material for: "Hearing the pupils’ voices through my own struggles”: A qualitative study of return to work among school counselors who are breast cancer survivors
Source: PLoS One. 2024 May 10;19(5):e0300396. doi: 10.1371/journal.pone.0300396 (PMC11086897; doi:10.1371/journal.pone.0300396)
Supplement: Attachment — Submitted filename: Response to reviewers.docx [file pone.0300396.s002.docx]

January 10, 2024

Prof. Michal Ptaszynski

Editor

*PLOS ONE*

Dear Professor Ptaszynski:

Submission of a Revision for Manuscript PONE-D-23-15124R1

Enclosed, please find our revised manuscript PONE-D-23-15124R1, titled “"Hearing the Pupils’ Voices Through My Own Struggles”: A Qualitative Study Of Return To Work Among School Counselors Who Are Breast Cancer Survivors” which we are submitting for possible publication in *PLOS ONE*.

We thank you and the reviewers for the useful comments, which have significantly improved our manuscript. We have attached a version of the paper that shows these changes, along with a final “clean” copy of the manuscript. Below, we delineate the specific modifications we made to address the reviewers’ concerns.

Sincerely,

Prof. Inbar Levkovich, Ph.D.

Faculty of Graduate Studies

Oranim Academic College of Education, Israel

Dr. Christina Signorelli

School of Clinical Medicine, UNSW Medicine & Health, Discipline of Paediatrics, UNSW Sydney, Kensington, NSW, Australia

Kids Cancer Centre, Sydney Children’s Hospital, NSW, Randwick, Australia

**Comments to the Author**

1. If the authors have adequately addressed your comments raised in a previous round of review and you feel that this manuscript is now acceptable for publication, you may indicate that here to bypass the “Comments to the Author” section, enter your conflict of interest statement in the “Confidential to Editor” section, and submit your "Accept" recommendation.

Reviewer #1: All comments have been addressed

Reviewer #3: All comments have been addressed

2. Is the manuscript technically sound, and do the data support the conclusions?

The manuscript must describe a technically sound piece of scientific research with data that supports the conclusions. Experiments must have been conducted rigorously, with appropriate controls, replication, and sample sizes. The conclusions must be drawn appropriately based on the data presented.

Reviewer #1: Yes

Reviewer #3: Yes

3. Has the statistical analysis been performed appropriately and rigorously?

Reviewer #1: N/A

Reviewer #3: N/A

4. Have the authors made all data underlying the findings in their manuscript fully available?

The [PLOS Data policy](http://www.plosone.org/static/policies.action#sharing) requires authors to make all data underlying the findings described in their manuscript fully available without restriction, with rare exception (please refer to the Data Availability Statement in the manuscript PDF file). The data should be provided as part of the manuscript or its supporting information, or deposited to a public repository. For example, in addition to summary statistics, the data points behind means, medians and variance measures should be available. If there are restrictions on publicly sharing data—e.g. participant privacy or use of data from a third party—those must be specified.

Reviewer #1: No

Thank you for the feedback. in accordance with the rules of the Ethics Committee and the Helsinki Committee and considering that our research involved personal interviews with women in cancer recovery, weregret that we are unable to share this information. Consequently, we have maintained the anonymity of the participants and have refrained from presenting their information in detail. We added our statement regarding data availability as follows:

**Availability of data and materials**: Data cannot be shared beyond individual quotations in the manuscript due to participants’ lack of consent for the publication of their full transcripts. The interviews contain sensitive data, and this decision aligns with the ethics committee of Oranim College. Please contact Miss Shaulov, administrative coordinator of the research authority in Oranim College for any further information: [research@oranim.ac.il](mailto:research@oranim.ac.il)

Reviewer #3: Yes

5. Is the manuscript presented in an intelligible fashion and written in standard English?

PLOS ONE does not copyedit accepted manuscripts, so the language in submitted articles must be clear, correct, and unambiguous. Any typographical or grammatical errors should be corrected at revision, so please note any specific errors here.

Reviewer #1: Yes

Reviewer #3: Yes

6. Review Comments to the Author

Please use the space provided to explain your answers to the questions above. You may also include additional comments for the author, including concerns about dual publication, research ethics, or publication ethics. (Please upload your review as an attachment if it exceeds 20,000 characters)

Reviewer #1: The authors have addressed the comments well. Some minor points for clarification:

1) "The participants of the current study expressed a desire to return to work but also its consequences." (page 19): the current phrasing suggests that participants expressed a desire for return to work consequences. Is this accurate?

Thanks for the feedback. We re-worded the sentence:

Page 19: “The participants of the current study expressed a desire to return to work.”
2) Under study limitations (page 21), suggest "results may not be transferrable to broader population" instead of 'generalized'. Also, it may be good to specify who constitutes the 'broader population'? Is it cancer survivor or breast cancer survivor population?
Thank you. Here is how we have revised the wording of the sentence:

Page 21: “The small size of the sample limits the generalizability of the findings to the broader breast cancer survivor population”.
3) Under study limitations (page 21), the description of the sampling method can be refined to specify the sampling bias introduced. For instance, this way of sampling likely excludes counsellors who are 'off the radar', not as actively involved in professional groups or social media.

Thank you for pointing this out.

Page 21: “In addition, this online design introduced bias in the sample towards population groups with digita literacy or access to digital resources and those who are more socially connected, at least in the virtual sense. Furthermore, the recruitment method through a Facebook support group may not fully represent all breast cancer survivors, which also poses a limitation."

Reviewer #3: The manuscript has been revised, addressing all the changes suggested by the reviewers. There are no suggestions.

7. PLOS authors have the option to publish the peer review history of their article ([what does this mean?](https://journals.plos.org/plosone/s/editorial-and-peer-review-process#loc-peer-review-history)). If published, this will include your full peer review and any attached files.
If you choose “no”, your identity will remain anonymous but your review may still be made public.

**Do you want your identity to be public for this peer review?** For information about this choice, including consent withdrawal, please see our [Privacy Policy](https://www.plos.org/privacy-policy).

Reviewer #1: No

Reviewer #3: **Yes:** Nicola Magnavita

We would like to express our gratitude once again to the reviewers for their generous assistance, which has significantly contributed to the improvement of our manuscript. We hope that the reviewers now find our manuscript suitable for publication in *PLOS ONE*.
